# The Association Between Diabetic Nephropathy and Triglyceride/Glucose Index and Triglyceride/High-Density Lipoprotein Cholesterol Ratio in Patients with Type 2 Diabetes Mellitus

**DOI:** 10.3390/jcm13226954

**Published:** 2024-11-18

**Authors:** Abbas Ali Tam, Feride Pınar Altay, Pervin Demir, Didem Ozdemir, Oya Topaloglu, Reyhan Ersoy, Bekir Cakır

**Affiliations:** 1 Department of Endocrinology and Metabolism, Faculty of Medicine, Ankara Yildirim Beyazit University, Ankara 06800, Turkey; sendidem2002@gmail.com (D.O.); oyasude@gmail.com (O.T.); reyhanersoy@hotmail.com (R.E.); drcakir@gmail.com (B.C.); 2 Department of Endocrinology and Metabolism, Ankara City Hospital, Ankara 06800, Turkey; fpaltay@gmail.com; 3 Department of Biostatistics and Medical Informatics, Faculty of Medicine, Ankara Yildirim Beyazit University, Ankara 06800, Turkey; pervin.demr@gmail.com

**Keywords:** diabetic nephropathy, triglyceride/glucose index, triglyceride/high-density lipoprotein cholesterol ratio, type 2 diabetes

## Abstract

**Background:** In this study, we aimed to investigate the relationship between diabetic nephropathy (DN) and triglyceride/glucose (TyG) index and triglyceride/high-density lipoprotein cholesterol ratio (Tg/HDL-C) as surrogate markers of insulin resistance. **Method:** Medical records of 15,378 individuals between February 2019 and May 2024 were examined. Serum glucose, Tg, HDL-C, HbA1c, estimated glomerular filtration rate (eGFR), and urine albumin/creatinine ratio (UACR) were evaluated and the TyG index and TG/HDL-C ratios were calculated for each individual. DN was defined as a UACR ≥ 30 mg/g and/or eGFR <60 mL/min/1.73 m^2^. **Results:** Of 10,714 patients, DN was detected in 3763 (35.1%). Females had 10% higher odds of developing DN compared to males. A TyG index at or above the determined cutoff point (9.58) indicated a risk of DN and the sensitivity and specificity values were 44.01% and 71.28%, respectively. The risk of DN was 1.95 times higher in individuals with a TyG index value of ≥9.58 compared to those with a TyG index <9.58. While the Tg/HDL ratio was significant in detecting DN in the univariate analysis (odds ratio (OR) 1.59; 95% confidence interval 1.46–1.73), this significance was not found in the multivariate analysis (OR 1.15; 95% confidence interval 0.94–1.40). **Conclusions:** A high TyG index is associated with DN in patients with type 2 diabetes and it might be a potential marker in predicting DN.

## 1. Introduction

Diabetic nephropathy (DN) is a common and severe microvascular complication of diabetes mellitus (DM) and an important cause of end-stage renal disease and mortality in diabetic patients [1]. Albuminuria increasing overtime and impairment in renal function are the clinical findings that indicate progressive kidney damage. Since DN has an insidious onset, slow progression, and nonspecific symptoms, the diagnosis is often delayed and it is commonly detected after the development of morbidities [2]. However, it is known that early detection allows for preventive treatments to be administered, slowing down the progression of the disease and reducing the development of complications [3].

One of the factors that affect the onset, progression, and prognosis of DN is insulin resistance (IR). It is a significant and independent risk factor for kidney disease [2,4,5,6,7]. It was shown that as the severity of IR increases, the risk of DN also increases [6,8]. Although hyperinsulinemic–euglycemic clamp is accepted as the gold standard method for the diagnosis of IR, its clinical use is limited due to its cost and complexity [9]. Homeostasis model assessment-IR (HOMA-IR) is another frequently used method for IR assessment, but it is time-consuming and expensive, and requires insulin measurement. Additionally, since some diabetic patients use insulin, the measurement may be inaccurate [2,6,9,10]. The triglyceride glucose index (TyG) is a simple, cost-effective, and accessible marker proposed to assess IR. It is calculated by fasting blood glucose and triglyceride. A correlation between TyG index and IR was proven in studies using hyperinsulinemic–euglycemic clamp or HOMA-IR as the reference methods [2,9,11]. The Tg/HDL-C ratio is another parameter that was suggested to be a better predictor of IR and cardiovascular disease than TG or HDL-C levels alone [12].

Considering that IR is a risk factor for chronic kidney disease (CKD) progression, promising markers, such as the TyG index that reflects IR, might help to detect CKD in type 2 DM in the early stages [13]. An increasing number of studies have been conducted on TyG to find a reliable and simple indicator for CKD in recent years. However, the number of studies conducted on patients with type 2 DM is few and they mostly include small sample sizes. The number of studies examining the relationship between the Tg/HDL-C ratio and DN is almost non-existent. In this study, we aimed to investigate the possible relationship between DN and the TyG index and TG/HDL-C ratio by conducting data mining in a large patient group.

## 2. Methods

The medical records of type 2 DM patients over the age of 18 who applied to the Endocrinology and Metabolism Diseases outpatient clinic of Ankara Bilkent City Hospital between February 2019 and June 2024, regardless of the reason for application and medication use, were examined. Although nearly 3% of patients with type 2 DM have overt nephropathy at diagnosis, it is rare to see DN in patients with type 1 diabetes before 10 years following diagnosis [3]. Therefore, in order to obtain a unique/homogenous data set, patients with type 1 DM and gestational DM were excluded from the study.

For each patient, age, gender, serum Tg, HDL-C, HbA1c, glucose, estimated glomerular filtration rate (eGFR), and urine albumin/creatinine ratio (UACR) values were recorded. The TyG index was calculated as the Ln [fasting TG (mg/dL) × fasting glucose (mg/dL)/2]. The Tg/HDL-C ratio was also calculated for each patient. Microalbuminuria was defined as a UACR > 30 mg/g and DN was defined as a UACR ≥ 30 mg/g and/or eGFR < 60 mL/min/1.73 m^2^. The Cockcroft-Gault formula described below was used to calculate eGFR [14].

Creatinine clearance (mL/min): [(140 − age) × body weight]/[plasma creatinine × 72] (× 0.85 if female).

## 3. Statistical Analysis

Data were summarized as the mean ± standard deviation or median (Quartile 1–Quartile 3) for quantitative variables, and as percentages for qualitative variables. In the comparison of numerical values between the patients with and without DN, the Mann–Whitney U test was employed, while Pearson correlation coefficients were used for categorical variables. Receiver operating characteristic curve (ROC) analysis was constructed to evaluate the discriminatory performance for DN presence according to the value of the area under the ROC (AUC). The optimal cutoff points were established based on the Youden index. An AUC value that did not include 0.50 was considered indicative of a significant ROC analysis result. A binary logistic regression univariate/multivariable analysis with DN categorized as a binary variable (presence or absence of DN) was used to evaluate the associations between the measured risk factors and DN. An odds ratio (OR) value greater than 1 indicated that the corresponding variable was a risk factor for DN. Additionally, the significance of the results was affirmed if the confidence interval did not include 0.50. The relationships between the TyG index and Tg/HDL ratio values and the variables used to define nephropathy, UACR and eGFR, were examined using Spearman’s rho correlation coefficient for the established groups. Statistical analyses were performed using the IBM SPSS Statistics version 21.0 (Armonk, NY, USA: IBM Corp). Statistical significance was defined by a *p*-value < 0.05.

## 4. Results

A total of 15,378 patients, consisting of 44.4% male and 55.6% female, with a mean age of 60.10 ± 12.12 years (range: 18–90), were included in the study. Among these patients, DN was identified in 3763 (35.1%) out of 10,714 patients with known diabetes status. Table 1 presents the characteristics of the patients stratified by groups (patients with and without DN). In men, DN was identified in 34.1%, while in women, the prevalence was 36.3% (*p* = 0.019). All other clinical variables between those with and without diabetic nephropathy were statistically significantly different (all *p* < 0.001). In patients with DN, UACR, glucose, triglyceride, HbA1c, Tg/HDL, and TyG index levels were higher compared to the patients without DN, while eGFR and HDL levels were lower.

The results of the ROC analysis and logistic regression model to evaluate the discriminatory performance of the variables for DN presence are presented in Table 2. The AUC was statistically significant for all the variables, and the performance metrics based on the determined cutoff points are presented. A TyG index at or above the determined cutoff point (9.58) indicated a risk of DN. Accordingly, the sensitivity and specificity values were 44.01% and 71.28%, respectively.

The odds ratio (OR) of 1.10 for gender (female/male) in the logistic regression model showed that females had 10% higher odds of developing DN compared to males. Since the OR was close to 1, this implies that gender may not be a strong independent risk factor for DN. Additionally, in individuals with a TyG index value of ≥9.58, the risk of DN was found to be 1.95 times greater compared to those with a TyG index < 9.58. Similarly, the likelihood of having DN was 2.08 times greater in individuals with HbA1c levels of ≥7% compared to individuals with HbA1c levels < 7%.

The Tg/HDL ratio had an OR of 1.59 for DN in the univariate analysis but became nonsignificant in the multivariate analysis, with an OR of 1.15, indicating that the association was no longer statistically significant when controlled for other factors.

**Figure 1:**
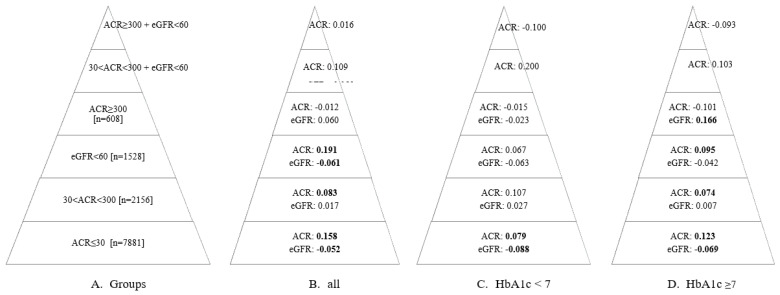
The relationship between TyG index values and ACR and eGFR values in the groups specified in Pyramid (**A**). Pyramid (**B**) shows the correlation coefficient (r) for the entire sample across each group. Pyramid (**C**) presents the correlation coefficient (r) for individuals with HbA1c values < 7 in each group. Pyramid (**D**) displays the correlation coefficient (r) for individuals with HbA1c values ≥7 in each group. The coefficients that were determined to be statistically significant (*p* < 0.05) are highlighted in bold. eGFR: estimated glomerular filtration rate; ACR: albumin/creatinine ratio.

The results of the analysis examining the relationship between the TyG index and the UACR and eGFR variables across different subgroups are presented in Figure 1. Accordingly, while the correlation coefficients in each group were relatively low, some groups showed statistically significant relationships (correlation coefficients that were determined to be statistically significant are highlighted in bold). In individuals with UACR ≤30 mg/g, the correlation coefficients were statistically significant in both the overall sample and the HbA1c groups (*p* < 0.05). A positive relationship was observed between the TyG index and UACR, while a negative relationship was noted with eGFR, both at low levels. The correlation coefficient between UACR and the TyG index for individuals with HbA1c levels ≥ 7% was 0.123, whereas for those with HbA1c levels < 7%, the coefficient was 0.079. In the group with ACR ≥ 300, no significant relationship was found between ACR and the TyG index (*p* > 0.05). For eGFR, there was no significant correlation in the overall sample or in individuals with HbA1c < 7% (*p* > 0.05). However, in individuals with HbA1c ≥ 7%, a significant positive relationship (r = 0.166) was observed between eGFR and the TyG index (*p* < 0.05).

## 5. Discussion

In the current study, we examined the relationship between the TyG index and Tg/HDL with DN, and a significant relationship was found between the TyG index and DN. A few studies have previously demonstrated an association between TyG index and DN. In a study including 682 hospitalized patients with type 2 DM, it was shown that a TyG index >9.66 was predictive for DN with a sensitivity of 61.7% and specificity of 76.0%, and it was associated with the development, but not the grade, of albuminuria. In addition, the TyG index had a greater ROC AUC score (AUC 0.67) for the identification of DN than HOMA2-IR (AUC 0.61) [7]. In another study, Lv et al. evaluated 1432 patients with type 2 DM at baseline and followed 424 of them for an average of 21 months (12–24). They reported that a higher risk of microalbuminuria and eGFR < 60 mL/min/1.73 m^2^ were associated with a higher TyG index. During follow-up, DN developed in 94 of 424 patients. A TyG index in the highest tertile at baseline posed a greater risk for developing DN than a TyG index in the lowest tertile [9]. Shang et al. found a non-linear relationship between the TyG index and the risk of newly diagnosed biopsy-proven DN. The threshold of TyG in that study was 9.07 (9.05–9.09) [5]. Ren et al. published a prospective study with 10,498 patients over the age of 45 and a meta-analysis including a total of 13 studies. There was a significant correlation between the TyG index and the risk of DN. Every 1-SD increase in the TyG index was associated with an 11% increased risk of DN. In the meta-analysis, the pooled relative risk of the highest TyG compared to the lowest TyG index quartile for DN was 1.47 (1.32–1.63) [15].

Altered lipid metabolism has an important pathogenic role in the development and progression of DN. Mu et al. examined the relationship between DN and four different parameters: TyG index, Tg/HDL ratio, Tg glucose-body mass index (TyG-BMI), and metabolic score for insulin resistance (METS-IR). Among these four parameters, the predictive value for the diagnosis of DN was highest with the TyG index and Tg/HDL, followed by METS-IR [10]. Wen et al. examined the possible relationship between visceral adiposity index (VAI) and microalbuminuria in patients with newly diagnosed type 2 DM and observed that microalbuminuria was significantly associated with VAI and TG/HDL. Each 1-SD increase in VAI and Tg/HDL increased the risk of microalbuminuria by 1.94 and 2.03 times, respectively [16]. In our study, there was a significant relationship between DN and both the TyG index and the Tg/HDL ratio in the univariate analysis. However, this significance disappeared for the Tg/HDL ratio in the multivariate analysis. As a cutoff, the risk of DN increased nearly by 2-fold when the TyG index was above 9.58. When we made a pyramid in terms of severity from UACR < 30 mg/g to UACR ≥ 300 mg/gr + eGFR < 60 mL/min/1.73 m^2^, there was a significant relationship between the TyG index and DN in the lower part of the pyramid. This may be due to the lower number of patients in the upper part of the pyramid. This might also suggest that the relationship between the TyG index and DN is significant in less severe periods of DN.

The pathophysiological mechanisms of the relationship between the TyG index and CKD or DN are not yet clear. Different contributors have been proposed to explain this association. Hyperinsulinemia causes renal vasodilation and subsequent glomerular hyperfiltration [17]. This hyperfiltration further causes nephron loss and leads to glomerular hypertension, which results with glomerular sclerosis and decline in renal function [18]. Additionally, IR activates the mitochondrial electron transport chain and increases reactive oxidative stress, which is a risk factor for renal tissue fibrosis [9]. Visceral adiposity is associated with the development of inflammation, oxidative stress, endothelial dysfunction, and atherosclerosis, which further causes glomerulosclerosis and tubulointerstitial fibrosis. The stimulation of the pro-inflammatory pathway and the activation of the renin–angiotensin–aldosterone system (RAAS) by increased adiposity cause hypertension and increase IR, which are important risk factors for renal injury [19].

It is difficult to treat DN, especially in later stages and end-stage renal disease. The early diagnosis of DN is essential to prevent the progression of the disease and to take timely precautions. For this, we need simple, reliable, and widely available indicators. Both our study and previous studies suggest that the TyG index might be used as a predictive tool to detect patients with a high risk of DN in clinical settings.

There are some limitations and strengths of our study. First of all, our study was a retrospective single-center study. Secondly, cross-sectional data of the patients were evaluated. The lack of follow-up data can be considered as another limitation of our study. It can be assumed that long-term follow-up data would provide more precise information about the utility and value of the TyG index. Another limitation was that we did not have information about the medications of the patients. On the other hand, to our knowledge, this is the largest-scale study examining the relationship between DN and the Tyg index and Tg/HDL ratio in patients with type 2 DM. Previous studies had various selection biases such as the inclusion of only male gender or patients over a certain age or hospitalized patients or patients with biopsy-proven renal disease. Therefore, a different cutoff was proposed for DN in each study. In order to provide a more precise cutoff value and to reveal whether the use of medications or interventions affecting the TyG index and Tg/HDL ratio will affect the onset and progression of DN, multicenter/large-scale studies in different populations are needed.

In conclusion, there was a significant relationship between the TyG index and DN in patients with type 2 DM. The TyG index can be used as a simple and cheap marker in the prediction of DN. A long-term follow-up will be required to find the clinical usefulness and significance of this index.

## Figures and Tables

**Table 1:** Characteristics of patients with and without diabetic nephropathy.

Variable	Overall		Non-DN		DN	
	Median (Q1−Q3)	Mean ± SD	Median (Q1–Q3)	Mean ± SD	Median (Q1–Q3)	Mean ± SD
Age	61 (53–68)	60.10 ± 12.12	60 (53–67)	58.95 ± 11.3	66 (58–73)	64.74 ± 11.54
Gender (%)						
Female	8550 (55.6%)		3851 (65.9%)		1996 (34.1%)	
Male	6828 (44.4%)		3100 (63.7%)		1767 (36. 3%)	
ACR (mg/g)	9.89 (4.56–31.98)	88.54 ± 383.17	6.34 (3.71–11.96)	8.77 ± 6.88	70.38 (37.1–196.26)	267.68 ± 655.92
≤30	7881 (74.0%)		6951 (93.1%)		515 (6.9%)	
30–300	2156 (20.3%)		0 (0.0%)		2156 (100.0%)	
≥300	608 (5.7%)		0 (0.0%)		608 (100.0%)	
eGFR (mL/min/1.73 m^2^)	90.51 (74.85–100.74)	86.86 ± 20.54	92.92 (81.53–101.45)	91.76 ± 14.58	69.31 (51.54–93.37)	71.05 ± 25.41
<60	1528 (11.0%)		0 (0.0%)		1528 (100.0%)	
≥60	12416 (89.0%)		6951 (77.2%)		2058 (22.8%)	
Glucose (mg/dL)	133 (107–180)	152.7 ± 63.73	131 (106–171)	147.95 ± 59.16	153 (118–211)	171.29 ± 70.66
Triglyceride (mg/dL)	151 (109–215)	182.44 ± 128.34	146 (106–204)	172.1 ± 111.49	164 (120–233.5)	201.71 ± 147.35
HbA1c (%)	7.4 (6.4–8.8)	7.83 ± 1.96	7.1 (6.3–8.5)	7.61 ± 1.83	8.0 (6.8–9.5)	8.36 ± 2.02
<7	3240 (40.6%)		1811 (76.0%)		571 (24.0%)	
≥7	4748 (59.4%)		2234 (60.4%)		1464 (39.6%)	
HDL (mg/dL)	44 (37–52)	45.59 ± 12.5	44.1 (37.8–53)	46.21 ± 12.28	42 (35.5–50)	43.92 ± 12.67
TG/HDL	3.46 (2.24–5.42)	4.61 ± 4.69	3.30 (2.15–5.07)	4.26 ± 4.11	3.92 (2.57–6.16)	5.31 ± 5.42
TyG index	9.25 (8.82–9.74)	9.31 ± 0.71	9.19 (8.78–9.66)	9.24 ± 0.67	9.45 (9–9.98)	9.52 ± 0.73

Values are expressed as the mean ± standard deviation, median (Quartile 1−Quartile 3), or frequency (percentage). According to the Mann–Whitney U test, all quantitative variables between those with and without diabetic nephropathy were statistically significantly different (all *p* < 0.001). The Pearson Chi-square test was used for categorical variables, and all *p*-values were <0.001, except for gender (*p* = 0.019). eGFR: estimated glomerular filtration rate; ACR: albumin/creatinine ratio; HDL: high-density lipoprotein; TG/HDL: triglyceride/high-density lipoprotein; TyG: triglyceride/glucose.

**Table 2:** Risk factor analysis for DN diagnosis: ROC, cutoff, and logistic regression results.

Variables	AUC (Lower–Upper)	Cutoff	Sen	Spe	Univariate OR (Lower–Upper)	Multivariate * OR (Lower–Upper)
Gender (F/M)					1.10 (1.02–1.91)	0.98 (0.87–1.12)
Age	0.647 (0.636–0.658)	>65	50.60	71.73	2.60 (2.39–2.82)	3.29 (2.91–3.72)
Glucose (mg/dL)	0.604 (0.592–0.615)	>150	51.72	64.95	1.99 (1.83–2.16)	1.50 (1.29–1.76)
Triglyceride (mg/dL)	0.571 (0.559–0.583)	≥160	52.58	57.20	1.48 (1.36–1.61)	1.19 (0.98–1.44)
HbA1c (%)	0.619 (0.604–0.634)	≥7	71.94	44.77	2.08 (1.85–2.33)	1.50 (1.29–1.74)
HDL (mg/dL)	0.561 (0.549–0.573)	≤40	43.96	64.55	1.43 (1.31–1.55)	1.27 (1.10–1.47)
TG/HDL	0.580 (0.568–0.592)	≥3.44	58.64	52.82	1.59 (1.46–1.73)	1.15 (0.94–1.40)
TyG index	0.610 (0.598–0.622)	≥9.58	44.01	71.28	1.95 (1.79–2.13)	1.25 (1.04–1.50)

* AUC: the area under the curve value with 95% confidence interval (C.I.) limits. The exclusion of 0.50 from the C.I. indicates that the AUC is statistically significant. OR: the odds ratio and 95% C.I. limits. If the C.I. includes 1, it suggests that the association may not be statistically significant. HDL: High-density lipoprotein; TG/HDL: Triglyceride/High-density lipoprotein; TyG: Triglyceride/Glucose.

## Data Availability

The datasets generated and analyzed in the present study are available upon reasonable request to the corresponding author.
